# Death, long-term nursing home placement, and impoverishment after recurrent myocardial infarction^☆^

**DOI:** 10.1016/j.ahjo.2021.100036

**Published:** 2021-07-30

**Authors:** Emily B. Levitan, Bharat Poudel, Lei Huang, Hong Zhao, Vera Bittner, Monika M. Safford, Elizabeth A. Jackson, Keri L. Monda, Paul Muntner

**Affiliations:** a Department of Epidemiology, University of Alabama at Birmingham, Birmingham, AL, USA; b Division of Cardiovascular Disease, Department of Medicine, University of Alabama at Birmingham, Birmingham, AL, USA; c Department of Medicine, Weill Cornell Medicine, New York, NY, USA; d Center for Observational Research, Amgen, Inc, Thousand Oaks, CA, USA

**Keywords:** Myocardial infarction, Mortality, Nursing home, Impoverishment

## Abstract

**Study objective:**

To determine whether recurrent myocardial infarction (MI) is associated with increased risk of mortality, long-term nursing home placement, and impoverishment.

**Design:**

Retrospective cohort study.

**Setting:**

United States Medicare program.

**Participants:**

Individuals age > 65 years with recurrent MI hospitalizations (*n* = 228,826) between January 1, 2007 and June 30, 2017 and controls with initial but not recurrent MI (*n* = 915,304).

**Main outcome measures:**

Death, nursing home placement, and impoverishment (Medicaid enrollment or subsidies for low-income and -resource individuals) through December 31, 2017.

**Results:**

In the recurrent MI and control cohorts, 47% and 41% of individuals were age > 80 years, respectively, and 56% of both cohorts were women. After 1 year, 48% of the recurrent MI cohort and 16% of the control cohort died, 9% and 7% experienced nursing home placement, and 4% and 2% experienced impoverishment. Multivariable-adjusted hazard ratios (95% confidence intervals) comparing the recurrent MI and control cohorts were 2.04 (2.03–2.06) for death, 0.89 (0.88–0.91) for nursing home placement, and 1.32 (1.28–1.36) for impoverishment.

**Conclusions:**

Older US adults with recurrent MI had higher risk of death and impoverishment than controls who had experienced an initial MI. Unadjusted, recurrent MI was associated with higher risk of nursing home placement; however, after adjusting for sociodemographic characteristics and comorbidities, individuals with recurrent MI had slightly lower risk of nursing home placement. Preventing recurrent MI may also reduce the risk of death and impoverishment among older US adults.

## Highlights

• Recurrent myocardial infarction may cause death and impoverishment.
• Comorbidities and other characteristics explain higher risk of nursing home placement.
• Secondary prevention may reduce the risk of impoverishment as well as death.

## 1 Introduction

In a study of adults hospitalized for acute medical conditions, 30% reported that they would rather die than live in a nursing home permanently [1]. Financial distress resulting from medical expenses can reduce well-being, health-related quality of life, medication adherence, and quality of care, a phenomenon that has been described as “financial toxicity” [2], [3], [4], [5]. Individuals with recurrent myocardial infarction (MI), events which are potentially preventable, may be particularly vulnerable to decreases in functional status and substantial out-of-pocket costs [6]. Cardiovascular disease, comorbidities, declines in functional capacity while hospitalized, and stress associated with hospitalization may result in need for ongoing nursing care. In addition to the distress caused by loss of autonomy, privacy, and a familiar home, long-term care in a nursing home is expensive, and very few United States (US) adults have long-term care insurance [7]. Acute MI is associated with both short- and long-term increases in medical costs [8], and US adults with cardiovascular disease and their families are more likely to experience financial hardship compared to individuals and their families without cardiovascular disease [9], [10]. Medical expenses may contribute to a large proportion of bankruptcies in the US, with cardiovascular disease being among the most costly conditions [11]. Even MI survivors with medical insurance in the US have copays for covered services and must pay for care that is not covered, often including long-term home or nursing home care. In this study, we compared the risk of death, long-term nursing home placement, and impoverishment among US Medicare beneficiaries who experienced a recurrent MI and controls from the same population who experienced an initial MI. Additionally, we investigated characteristics associated with death, nursing home placement, and impoverishment following recurrent MI.

## 2 Methods

### 2.1 Study population

Medicare is a US federal health insurance program for adults aged 65 years and older and individuals with disabilities or end-stage renal disease. Fee-for-service Part A (hospital services), Part B (outpatient and other medical services), and Part D (prescription drugs) claims are available for research purposes. Alternatively, beneficiaries may opt for privately administered capitated coverage, referred to as Medicare Advantage. This study includes individuals with fee-for-service Medicare coverage. Data to replicate this study are available from the US Centers for Medicare and Medicaid Services. Protocols and statistical code are available from the authors. The Centers for Medicare and Medicaid Services Privacy Board approved this research, and the Institutional Review Board at the University of Alabama at Birmingham approved the current analysis with a waiver of informed consent.

We identified the population for this study from Medicare beneficiaries with fee-for-service coverage who experienced a hospitalization for MI between January 1, 2007 and June 30, 2017. We defined MI hospitalizations as overnight hospitalizations with an MI discharge diagnosis code (International Classification of Diseases, 9th Revision [ICD9], codes 410.x0 or 410.x1 or International Classification of Diseases, 10th Revision [ICD10], codes I21.xx or I22.xx) in any position and hospital stay ≤30 days. Beneficiaries who had a second MI hospitalization between January 1, 2007 and June 30, 2017 were included in the recurrent MI cohort (Supplementary Fig. 1). Follow-up began on the admission date of the recurrent MI hospitalization, referred to as the index date. Because claims were needed to assess MI events and beneficiary characteristics, we restricted the population to beneficiaries who had continuous Part A, B, and D coverage without Medicare Advantage between the initial and recurrent MI hospitalizations and for a minimum of 365 days prior to the recurrent MI hospitalization, were > 65 years of age on the index date, and lived in the US for 365 days prior to the index date. We additionally identified a control cohort without recurrent MI. For each individual in the recurrent MI cohort, we identified 4 beneficiaries who met all of the inclusion criteria listed above, matched on calendar year of initial MI. To be eligible as a control, the beneficiary had to be alive without a recurrent MI hospitalization for at least as much time following their initial MI as the beneficiary with recurrent MI to which they were matched. The index date for patients in the control cohort was set as the same number of days following their initial MI as their match in the recurrent MI cohort (Supplementary Fig. 2). Beneficiaries selected in the control cohort could go on to experience recurrent MI.

### 2.2 Beneficiary characteristics

We used Medicare enrollment information to assess age, sex, race/ethnicity, eligibility for Medicaid or the Part D low-income subsidy (prevalent impoverishment), and residential ZIP code linked to 2006–2010 American Community Survey data from the US Census Bureau to obtain area-level median income. We used diagnosis and procedure codes documented on claims from the 365 days prior to the index date (admission date for the recurrent MI cohort or matched date for the control cohort) to assess documented alcohol and tobacco use, prevalent nursing home residence [12], history of diabetes mellitus, chronic kidney disease (CKD), stroke, heart failure, peripheral artery disease (PAD), depression, and dementia, and healthcare utilization including cardiologist care, hospitalization year prior to the index date, use of statins, non-statin lipid lowering therapy, beta-blockers, and antiplatelet agents. We used claims from the 365 days prior to the index date through 30 days following the index date to assess cardiac rehabilitation. We calculated a claims-based frailty score with a range of 0–1 using diagnosis and procedure codes [13], [14]. We categorized the frailty score into robust (<0.15), pre-frail (0.15- < 0.25), mildly frail (0.25- < 0.35), and moderate-to-severely frail (≥0.35), as previously described [13]. We used the initial MI hospitalization to classify the event as primary or secondary diagnosis of MI and to assess coronary revascularization procedures.

### 2.3 Outcomes

We abstracted date of death from Medicare enrollment files. Medicare receives information on beneficiary deaths from the Social Security Administration, claims submitted by healthcare providers, and proxy reports. As in prior work [15], we operationalized long-term nursing home placement using physician evaluation and management codes for care provided in a nursing home setting during periods when the beneficiary was not eligible for Medicare skilled nursing benefits to distinguish between long-term residence in a nursing home and short post-hospitalization rehabilitation stays [12]. Impoverishment was operationalized as new enrollment in Medicaid or Medicare Part D low-income premium subsidies—health insurance programs for low-income and low-resource individuals [15]. Beneficiaries who were nursing home residents prior to the index date were excluded from analyses of incident long-term nursing home placement, and beneficiaries who were enrolled in Medicaid or the Medicare Part D low-income subsidy prior to the index date were excluded from analyses of incident impoverishment. Beneficiaries were followed from admission date for the recurrent MI cohort or matched date for the control cohort (i.e., the index date) through the date of the outcome of interest, loss of fee-for-service coverage, mortality, or December 31, 2017, whichever occurred first.

### 2.4 Statistical analysis

We described characteristics of the recurrent MI and control cohorts using frequencies and percentages. We calculated the cumulative incidence of death, long-term nursing home placement, and impoverishment for the recurrent MI and control cohorts using the Kaplan-Meier method. We used Cox proportional hazard models with the Breslow method for tie handling to estimate hazard ratios (HR) and 95% confidence intervals (CI) for death, long-term nursing home placement, and impoverishment, separately, among beneficiaries in the recurrent MI cohort compared to controls. As the Medicaid policies related to eligibility, including “spending-down” savings to obtain eligibility, and benefits vary by state [16], we allowed the baseline hazards (i.e., the rates of death, long-term nursing home placement, and impoverishment) to vary across states. The first model was unadjusted. The second model was adjusted for age, sex, and race/ethnicity. The third model was adjusted for variables in the second model and area-level median income, alcohol and tobacco use, primary versus secondary diagnosis position for first MI, coronary revascularization, history of diabetes mellitus, CKD, stroke, heart failure, PAD, depression, dementia, frailty, cardiologist care, any hospitalization within the past year, statin use and intensity (no statin use, use of low/moderate-intensity statins, use of high-intensity statins), non-statin lipid lowering therapy, use of beta-blockers, antiplatelet agents, cardiac rehabilitation, prevalent nursing home residence (for the mortality and impoverishment outcomes), and prevalent impoverishment (for the mortality and long-term nursing home placement outcomes). Because log(−log(survival)) plots suggested violation of the proportional hazards assumption, particularly for death, we constructed models that included interactions between recurrent MI and follow-up time (0–15, 16–30, 31–365, 366–730, and ≥ 731 days of follow-up). Because these models are computationally intensive, we selected a 20% random sample of the study population for this analysis. Among beneficiaries with recurrent MI, we used Cox proportional hazards models, adjusted as described for Model 3 above, to estimate the associations of patient demographics and clinical characteristics with death, long-term nursing home placement, and impoverishment, separately. In sensitivity analyses, we restricted the study population to beneficiaries who had a primary discharge diagnosis of MI during the initial MI hospitalization. All data management and analyses were conducted using SAS 9.4 (SAS Institute, Cary, NC).

## 3 Results

### 3.1 Study population

Among 228,826 Medicare beneficiaries with recurrent MI and 915,304 matched controls eligible for the mortality analysis, 214,531 with recurrent MI and 857,973 controls were eligible for the analyses of incident nursing home placement, and 119,182 with recurrent MI and 559,425 matched controls were eligible for the analyses of incident impoverishment. Compared to controls, individuals who experienced recurrent MI were older, less likely to be white, were frailer, were less likely to have a coronary revascularization procedure and more likely to have a secondary diagnosis of MI during the initial MI hospitalization (Table 1). Additionally, beneficiaries with recurrent MI were more likely to have a history of diabetes, CKD, stroke, heart failure, PAD, depression, and dementia.

**Table 1:** Characteristics of Medicare beneficiaries with and without recurrent myocardial infarction.

	Control cohort with initial MI	Recurrent MI cohort
Age, n (%)		
66–70 years	160,615 (17.5%)	35,920 (15.7%)
71–75 years	194,290 (21.2%)	42,677 (18.7%)
76–80 years	184,195 (20.1%)	43,112 (18.8%)
81–85 years	168,063 (18.4%)	43,300 (18.9%)
≥86 years	208,141 (22.7%)	63,817 (27.9%)
Sex, n (%)		
Male	406,587 (44.4%)	101,041 (44.2%)
Female	508,717 (55.6%)	127,785 (55.8%)
Race/Ethnicity, n (%)		
White	788,954 (86.2%)	189,283 (82.7%)
Black	71,466 (7.8%)	23,794 (10.4%)
Hispanic/Latino	19,488 (2.1%)	6030 (2.6%)
Asian	16,734 (1.8%)	4910 (2.1%)
Other	18,662 (2.0%)	4809 (2.1%)
Area-level median income, n (%)		
Not known	15,883 (1.7%)	4065 (1.8%)
<$30,000	52,108 (5.7%)	15,833 (6.9%)
$30,000 - $44,999	319,480 (34.9%)	82,480 (36.0%)
$45,000 - $59,999	262,445 (28.7%)	64,199 (28.1%)
$60,000 - $74,999	130,290 (14.2%)	31,423 (13.7%)
≥$75,000	135,098 (14.8%)	30,826 (13.5%)
Alcohol use, n (%)	1774 (0.2%)	616 (0.3%)
Tobacco use, n (%)	249,127 (27.2%)	71,909 (31.4%)
Diagnosis position for initial MI, n (%)		
Primary	642,993 (70.2%)	154,737 (67.6%)
Secondary	272,311 (29.8%)	74,089 (32.4%)
Coronary revascularization during initial MI hospitalization, n (%)	418,894 (45.8%)	74,592 (32.6%)
History of diabetes mellitus, n (%)	380,164 (41.5%)	127,997 (55.9%)
History of chronic kidney disease, n (%)	356,126 (38.9%)	154,526 (67.5%)
History of stroke, n (%)	89,982 (9.8%)	34,450 (15.1%)
History of heart failure, n (%)	405,359 (44.3%)	172,465 (75.4%)
History of peripheral artery disease, n (%)	140,625 (15.4%)	64,212 (28.1%)
History of depression, n (%)	325,795 (35.6%)	93,856 (41.0%)
History of dementia, n (%)	126,878 (13.9%)	45,930 (20.1%)
Frailty score, n (%)		
Robust	70,628 (7.7%)	6281 (2.7%)
Pre-frail	383,764 (41.9%)	69,378 (30.3%)
Mildly frail	277,601 (30.3%)	83,803 (36.6%)
Moderate-to-severely frail	183,311 (20.0%)	69,364 (30.3%)
Cardiologist care, n (%)	566,739 (61.9%)	137,462 (60.1%)
Any hospitalization within the past year, n (%)	634,409 (69.3%)	176,369 (77.1%)
Statin use and intensity, n (%)		
No statins	219,032 (23.9%)	59,760 (26.1%)
Low to moderate intensity statins	496,310 (54.2%)	111,006 (48.5%)
High-intensity statins	199,962 (21.8%)	58,060 (25.4%)
Non-statin lipid lowering treatment, n (%)	113,786 (12.4%)	30,278 (13.2%)
Use of beta-blockers, n (%)	739,635 (80.8%)	188,193 (82.2%)
Use of antiplatelet agents, n (%)	442,744 (48.4%)	115,090 (50.3%)
Cardiac rehabilitation, n (%)	32,367 (3.5%)	11,786 (5.2%)
Prevalent nursing home residence, n (%)	57,331 (6.3%)	14,295 (6.2%)
Prevalent impoverishment, n (%)	355,879 (38.9%)	109,644 (47.9%)

Note: MI = myocardial infarction.

### 3.2 Recurrent MI and death, long-term nursing home placement, and impoverishment

**Fig. 1:**
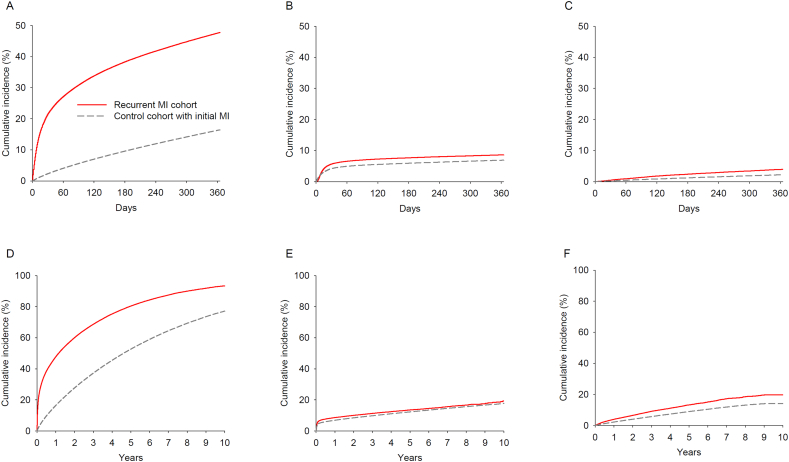
Cumulative incident of death (A and D), long-term nursing home placement (B and E), and impoverishment (C and F) after up to 1 year and 10 years of follow-up among Medicare beneficiaries with and without recurrent MI.

Among the recurrent MI cohort, there were 161,616 deaths, 17,815 cases of incident long-term nursing home placement, and 6071 cases of incident impoverishment over a median follow-up of 0.9 years (interquartile range 0.1–2.3 years). Among the matched control cohort, there were 402,478 deaths, 81,259 cases of incident long-term nursing home placement, and 30,770 cases of incident impoverishment over a median follow-up of 2.2 years (interquartile range 1.0–4.2 years). Individuals with recurrent MI had a higher incidence of death, long-term nursing home placement, and impoverishment than controls (Fig. 1). The cumulative incidence of death at 1 year was 47.7% and 16.4% in the recurrent MI and control cohorts, respectively. The cumulative incidence of long-term nursing home placement at 1 year was 8.6% and 6.9% in the recurrent MI and control cohorts, respectively. The cumulative incidence of impoverishment at 1 year was 4.0% and 2.2% in the recurrent MI and control cohorts, respectively.

**Fig. 2:**
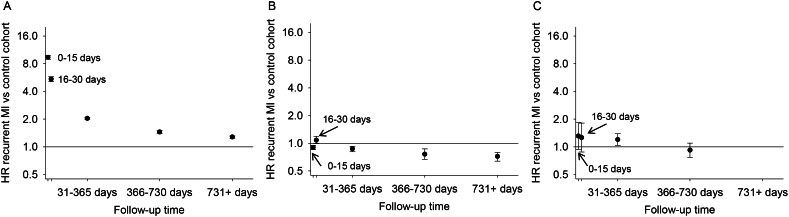
Association of recurrent MI with death (A), long-term nursing home placement (B), and impoverishment (C) over time since recurrent MI among Medicare beneficiaries. Adjusted for age, sex, race/ethnicity, area-level median income, alcohol and tobacco use, primary versus secondary diagnosis position for initial MI, coronary revascularization during hospitalization for initial MI, history of diabetes mellitus, chronic kidney disease, stroke, heart failure, peripheral artery disease, depression, and dementia, frailty, cardiologist care, cardiac rehabilitation, any hospitalization within the past year, statin use and intensity (i.e., no statin use, use of low/moderate-intensity statins, use of high-intensity statins), non-statin lipid lowering therapy, use of beta-blocker, antiplatelet agents, prevalent nursing home residence (mortality and impoverishment outcomes), and prevalent impoverishment (mortality and long-term nursing home placement outcomes), baseline hazard allowed to vary across states. The multivariable-adjusted HR for impoverishment after 731 days of follow-up could not be estimated because of the small number of cases in the recurrent MI cohort.

The unadjusted HR for mortality comparing the recurrent MI and matched control cohorts was 2.68 (95% CI 2.67–2.70) (Table 2). The mortality rate among individuals with recurrent MI remained double that among matched controls after adjusting for demographics, comorbidities, and healthcare utilization (HR 2.04, 95% CI 2.03–2.06). The crude HR for long-term nursing home placement was 1.18 (95% CI 1.16–1.19), but the association was attenuated when adjusting for demographics, and after adjusting for comorbidities, the individuals with recurrent MI had lower rates of long-term nursing home placement than individuals in the matched control cohort (HR 0.89, 95% CI 0.88–0.91). The HR for impoverishment was 1.58 (95% CI 1.53–1.62) comparing individuals with recurrent MI to matched controls in an unadjusted model and 1.32 (95% CI 1.28–1.36) after adjusting for demographics, comorbidities, and healthcare utilization. The HRs for death and impoverishment were highest early during follow-up and decreased over time since recurrent MI (Fig. 2 and Supplementary Table 1). For long-term nursing home placement, the HR was highest at days 16–30 of follow-up.

**Table 2:** Association of recurrent MI with death, long-term nursing home placement, and impoverishment among Medicare beneficiaries.

	Control cohort with initial MI	Recurrent MI cohort
Death		
Event/person-years	402,478/2,598,651	161,616/358,773
Rate per 1000 person-years (95% CI)	154.9 (154.4–155.4)	450.5 (448.3–452.7)
HR (95% CI)		
Model 1^a^	1 (reference)	2.68 (2.67–2.70)
Model 2^b^	1 (reference)	2.61 (2.60–2.63)
Model 3^c^	1 (reference)	2.04 (2.03–2.06)
Long-term nursing home placement		
Event/person-years	81,259/2,356,383	17,815/324,719
Rate per 1000 person-years (95% CI)	34.5 (34.2–34.7)	54.9 (54.1–55.7)
HR (95% CI)		
Model 1^a^	1 (reference)	1.18 (1.16–1.19)
Model 2^b^	1 (reference)	1.11 (1.09–1.13)
Model 3^c^	1 (reference)	0.89 (0.88–0.91)
Impoverishment^d^		
Event/person-years	30,770/1,613,408	6071/189,696
Rate per 1000 person-years (95% CI)	19.1 (18.9–19.3)	32.0 (31.2–32.8)
HR (95% CI)		
Model 1^a^	1 (reference)	1.58 (1.53–1.62)
Model 2^b^	1 (reference)	1.51 (1.47–1.56)
Model 3^c^	1 (reference)	1.32 (1.28–1.36)

Note: CI = confidence interval, HR = hazard ratio, MI = myocardial infarction.

a Unadjusted, baseline hazard allowed to vary across states.

b Adjusted for age, sex, and race/ethnicity, baseline hazard allowed to vary across states.

c Adjusted for age, sex, race/ethnicity, area-level median income, alcohol and tobacco use, primary versus secondary diagnosis position for initial MI, coronary revascularization during hospitalization for initial MI, history of diabetes mellitus, chronic kidney disease, stroke, heart failure, peripheral artery disease, depression, and dementia, frailty, cardiologist care, cardiac rehabilitation, any hospitalization within the past year, statin use and intensity (i.e., no statin use, use of low/moderate-intensity statins, use of high-intensity statins), non-statin lipid lowering therapy, use of beta-blocker, antiplatelet agents, prevalent nursing home residence (mortality and impoverishment outcomes), and prevalent impoverishment (mortality and long-term nursing home placement outcomes), baseline hazard allowed to vary across states.

d Defined as new Medicaid or Medicare Part D subsidy enrollment.

### 3.3 Characteristics associated with death, long-term nursing home placement, and impoverishment

**Fig. 3:**
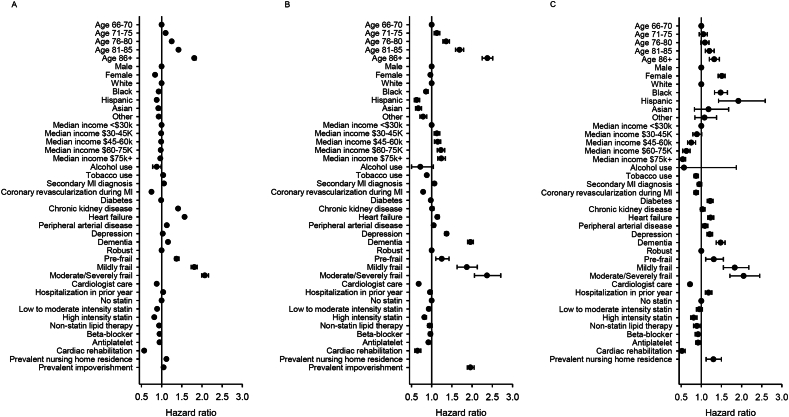
Characteristics associated with death (A), long-term nursing home placement (B), and impoverishment (C) among Medicare beneficiaries with recurrent myocardial infarction Hazard ratios calculated using Cox proportional hazards models include all characteristics listed. Baseline hazards were allowed to vary by state.

Among individuals with recurrent MI, older age, stroke, heart failure, depression, dementia, and higher frailty score were associated with a higher risk of death, long-term nursing home placement, and impoverishment (Fig. 3 and Supplementary Table 2). Revascularization during the initial MI, cardiologist care, use of statins, non-statin lipid lowering treatment, and antiplatelet agents, and cardiac rehabilitation were associated with a lower risk of death, long-term nursing home placement, and impoverishment. The associations for other characteristics varied across outcomes. For example, women had a lower risk of death but a higher risk of impoverishment than men. Black and Hispanic individuals had a lower risk of long-term nursing home placement but a higher risk of impoverishment than White individuals. Individuals living in areas with higher median incomes had lower risk of death and impoverishment but a higher risk of long-term nursing home placement than individuals living in lower income areas.

### 3.4 Sensitivity analysis

When the analysis was restricted to those whose initial MI was the primary discharge diagnosis, the associations of recurrent MI with death, long-term nursing home placement, and impoverishment and the associations of patient characteristics with these outcomes were similar to the primary analysis (Supplementary Tables 3–5).

## 4 Discussion

Among older US adults >65 years of age, individuals with recurrent MI had a higher risk of death, long-term nursing home placement, and impoverishment compared to controls who experienced only an initial MI. Long-term nursing home placement and impoverishment are important outcomes to patients and their families, but these outcomes have not been as extensively researched among individuals with cardiovascular disease compared to recurrent cardiovascular events and mortality. Recurrent MI remained associated with higher risk of death and impoverishment in models adjusted for demographics and comorbidities. However, after adjustment, individuals with recurrent MI had slightly lower risk of long-term nursing home placement than individuals with an initial MI only. There are multiple established pharmacologic and non-pharmacologic approaches to reduce risk of recurrent cardiovascular events among older adults, although the potential for harms from these therapies are greater in older compared to younger adults [6]. The findings of the current study suggest that prevention of recurrent MI might reduce impoverishment, in addition to the known benefits on mortality.

Although administrative claims data do not contain direct measurements of quality of life or other patient-reported outcomes, claims data can allow for assessment of other outcomes of importance to patients including long-term nursing home placement and new enrollment in programs for impoverished individuals, as in this study. The long-term nursing home placement and impoverishment outcomes are interrelated; many older adults living in long-term care settings enroll in Medicaid, a state/federal health insurance program for economically disadvantaged individuals, after depleting their assets paying for residential care [16]. In both the recurrent MI cohort and matched controls who experienced an initial MI, death was by far the most common of the outcomes examined, with nearly half of those with recurrent MI and 1 in 6 of those with an initial MI dying in the first year of follow-up. Because of the high rates of mortality in this population of older adults, we considered competing risk analyses. However, measures of association calculated using these methods represent a mixture of the associations with the outcome of interest and the competing risk, complicating interpretation [17].

To our knowledge, no other studies have specifically assessed the association of recurrent MI with long-term nursing home placement and impoverishment. However, there have been studies of cardiovascular disease in nursing home residents and the role of cardiovascular disease events on financial status. Approximately 38% of nursing home residents in the US have cardiovascular disease [18]. In one study of older adults, being hospitalized in the prior month was associated with a 60-fold higher rate of becoming disabled [19], a major risk factor for nursing home placement. In that study population, cardiovascular disease was the most common reason for hospitalization [20], and disability developed after 23% of hospitalizations for cardiovascular disease [19]. However, these studies did not identify whether cardiovascular disease per se resulted in the need for long-term nursing care. In the current study, recurrent MI was associated with a higher risk of long-term nursing home placement in unadjusted analyses but after multivariable adjustment, individuals with recurrent MI had a lower hazard of long-term nursing home placement than the control population. Older age, history of dementia, frailty, and prevalent impoverishment were strong risk factors for incident nursing home placement. Cardiac care services were associated with a lower rate of nursing home placement which probably reflects a combination of more intensive cardiac care being provided to lower risk individuals [21] and effects of the therapies reducing the need for nursing care.

In the US and other countries, studies have shown that cardiovascular disease events can lead to catastrophic health spending and impoverishment [22], [23]. Cardiovascular disease accounts for more medical expenditures than any other category of diseases among adults in the US [24]. According to one study, medical expenses contributed to more than half of US bankruptcies, with cardiovascular disease among the most costly conditions [11]. However, approaches to determine whether bankruptcy is related to medical expenses are controversial [25]. In the US, most households filing for bankruptcies have health insurance [11], and reductions in the number of people who were uninsured did not have a substantial effect on rates of bankruptcy [26]. Among US adults with cardiovascular disease aged less than 65, having health insurance was associated with less financial hardship; however, even among insured individuals, 41% of those without comorbid diabetes and 48% of those with comorbid diabetes reported difficulty paying medical bills [10]. In the current study of insured older adults, recurrent MI, older age, history of dementia, frailty, and prevalent nursing home residence were associated with higher risk for impoverishment, while cardiac care was associated with lower risk of impoverishment.

Strengths of this study include a very large study population of older adults with MI from across the US which allowed for precise estimates of the association of recurrent MI with death, long-term nursing home placement, and impoverishment and for examination of characteristics associated with these outcomes among older adults with recurrent MI. However, there are also several important limitations. We included only older US adults who were fee-for-service Medicare beneficiaries. Given differences in how medical care is delivered and financed, the findings, particularly for impoverishment, are unlikely to apply to older adults in other countries. Additionally, the generalizability to younger adults is unclear. We were unable to capture information on care arrangements other than nursing homes, such as home health care services, residential care communities, or informal care by family and friends [18]. Medicaid programs in some states support these alternatives to long-term nursing home placement [14]. Impoverishment was operationalized as new enrollment in Medicaid or Medicare Part D subsidies for prescription drug coverage. Although these programs support medical care for individuals with low income and low resources, relying on enrollment in Medicaid or Medicare Part D subsidies likely underestimates the financial burden associated with recurrent MI. Medicaid programs are administered by the states, and eligibility and services covered vary [16]. Of particular importance, states govern the process by which older adults “spend-down” their resources paying for long-term care and become eligible for Medicaid, leading to heterogeneity across states [16]. Additionally, individuals and their families vary in their knowledge of and access to benefits for which they are eligible. Because we matched individuals in control cohort on calendar year of initial MI and required survival without a recurrent MI hospitalization for at least as long as the individual in the recurrent MI cohort, characteristics of the recurrent MI and control groups may be more similar than would be observed among all people with MI. We were not able to exclude the possibility that the initial MI observed in this study was not the individual's first event; our study design did not detect MIs that occurred prior to 2007 and those occurring prior to an individual becoming Medicare eligible. We adjusted models for beneficiary demographics and health conditions, but claims data provide limited information on physiology and illness severity, and residual confounding may remain.

## 5 Conclusions

In summary, Medicare beneficiaries with recurrent MI had a higher risk of death, long-term nursing home placement, and impoverishment than controls who only experienced an initial MI. In adjusted models, the rate of death and impoverishment remained higher among individuals with recurrent MI. This study suggests that preventing recurrent MI may also reduce the risk of impoverishment and death among older US adults.

## Funding

This work was supported by an academic collaboration between 10.13039/100008333University of Alabama at Birmingham, 10.13039/100007273Weill Cornell Medicine, and 10.13039/100002429Amgen Inc. The funders provided comments on the design and interpretation of this work. The academic authors conducted all analyses and maintained the rights to publish this manuscript.

## CRediT authorship contribution statement

**Emily B. Levitan:** Conceptualization, Methodology, Writing – original draft, Visualization. **Bharat Poudel:** Investigation, Project administration, Writing – review & editing. **Lei Huang:** Software, Formal analysis, Data curation, Writing – review & editing. **Hong Zhao:** Software, Writing – review & editing. **Vera Bittner:** Conceptualization, Writing – review & editing. **Monika M. Safford:** Conceptualization, Writing – review & editing. **Elizabeth A. Jackson:** Conceptualization, Writing – review & editing. **Keri L. Monda:** Conceptualization, Writing – review & editing, Funding acquisition. **Paul Muntner:** Conceptualization, Writing – review & editing, Funding acquisition, Methodology.

## Declaration of competing interest

EBL reports research funding from Amgen and serving as a consultant for a Novartis funded research project. VB reports research funding from Sanofi, AstraZeneca, DalCor, Esperion, and Bayer, and consulting for Sanofi. MMS reports research funding from Amgen. EAJ reports research funding from Amgen and consulting income from McKesson and UpToDate. KLM reports Amgen employment and stock ownership. PM reports research funding from Amgen. The other authors have no interests to report.

## Appendix A Supplementary data

Supplementary material Image 1
