# Supplementary material for: Death, long-term nursing home placement, and impoverishment after recurrent myocardial infarction
Source: Am Heart J Plus. 2021 Jul 30;7:100036. doi: 10.1016/j.ahjo.2021.100036 (PMC10978126; doi:10.1016/j.ahjo.2021.100036)
Supplement: Supplementary file 1 — Supplementary material [file mmc1.docx]

**SUPPLEMENTAL MATERIAL: Death, long-term nursing home placement, and impoverishment after recurrent myocardial infarction**

**Supplementary Figure 1.** Flow diagram for identification of Medicare beneficiaries with recurrent myocardial infarction


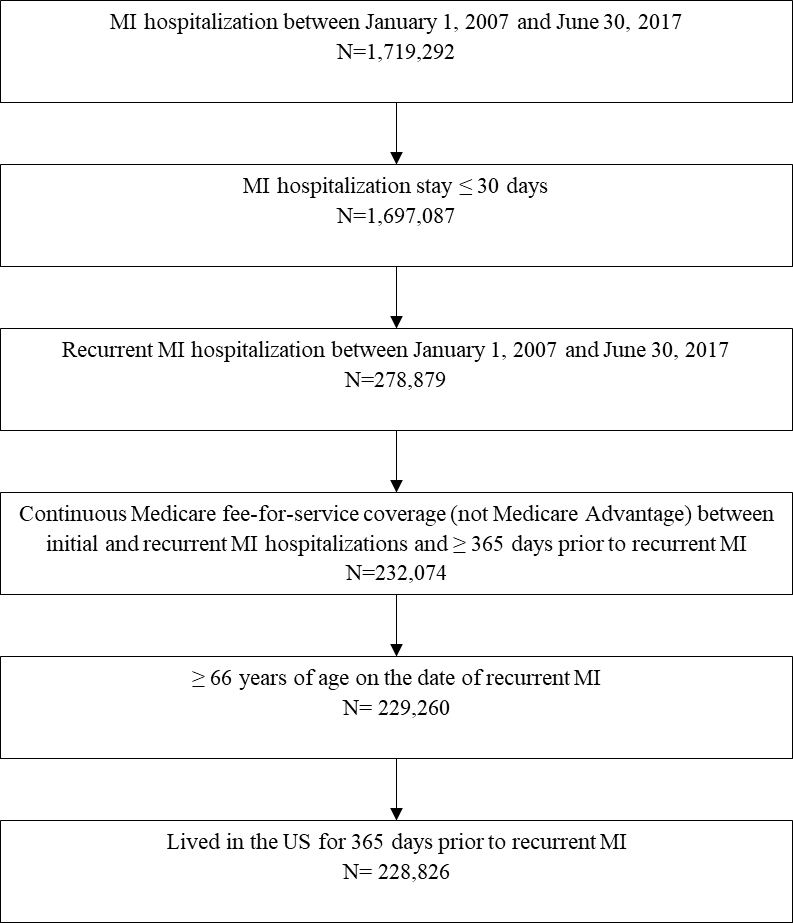


Abbreviation: MI, myocardial infarction

**Supplementary Figure 2.** Example of selection of controls matched on calendar year of initial myocardial infarction (MI) and time since initial MI


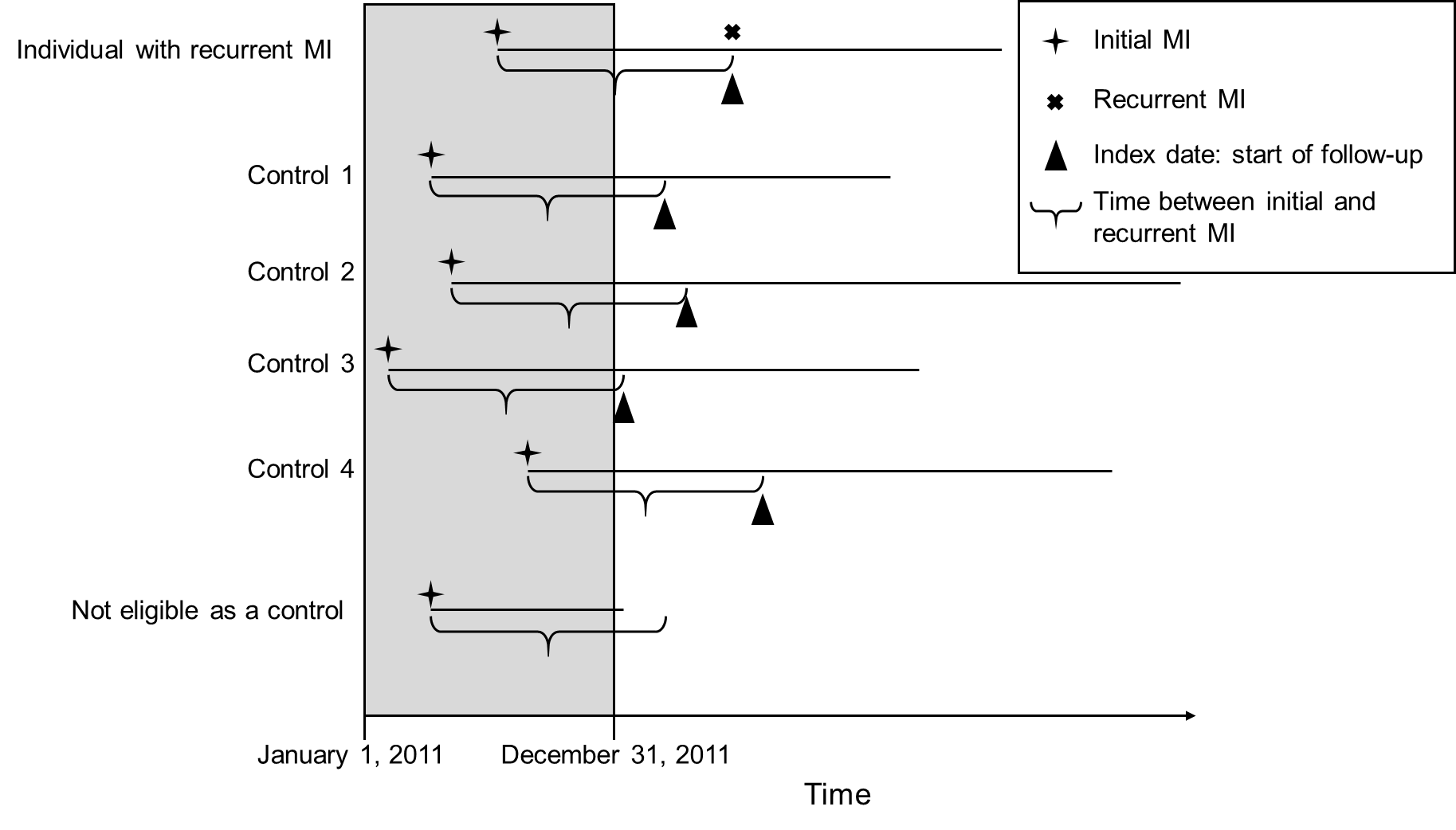


**Supplementary Table 1.** Association of recurrent MI with death, long-term nursing home placement, and impoverishment over time since recurrent MI among Medicare beneficiaries

|  | **Recurrent MI cohort compared to control cohort with initial MI** | | | | |
| --- | --- | --- | --- | --- | --- |
|  | **0-15 days** | **16-30 days** | **31-365 days** | **366-730 days** | **≥ 731days** |
| *Death* |  |  |  |  |  |
| HR (95% CI) |  |  |  |  |  |
| Model 1^a^ | 13.29 (12.68-13.94) | 7.46 (7.04-7.90) | 2.68 (2.62-2.74) | 1.89 (1.83-1.95) | 1.65 (1.60-1.69) |
| Model 2^b^ | 12.58 (11.99-13.19) | 7.15 (6.75-7.58) | 2.61 (2.56-2.67) | 1.85 (1.79-1.91) | 1.61 (1.57-1.66) |
| Model 3^c^ | 9.39 (8.95-9.85) | 5.45 (5.14-5.78) | 2.04 (1.99-2.08) | 1.45 (1.40-1.50) | 1.28 (1.24-1.31) |
| *Long-term nursing home placement* | | |  |  |  |
| HR (95% CI) |  |  |  |  |  |
| Model 1^a^ | 1.24 (1.17-1.31) | 1.44 (1.31-1.58) | 1.12 (1.05-1.21) | 0.97 (0.85-1.11) | 0.91 (0.81-1.02) |
| Model 2^b^ | 1.14 (1.08-1.20) | 1.35 (1.23-1.48) | 1.07 (1.00-1.15) | 0.93 (0.81-1.06) | 0.87 (0.78-0.98) |
| Model 3^c^ | 0.90 (0.85-0.95) | 1.08 (0.99-1.19) | 0.87 (0.81-0.93) | 0.76 (0.67-0.87) | 0.72 (0.64-0.80) |
| *Impoverishment*^d^ |  |  |  |  |  |
| HR (95% CI) |  |  |  |  |  |
| Model 1^a^ | 2.18 (1.59-2.98) | 2.03 (1.44-2.87) | 1.85 (1.68-2.03) | 1.39 (1.21-1.60) | 1.44 (1.29-1.61) |
| Model 2^b^ | 2.02 (1.48-2.77) | 1.91 (1.35-2.69) | 1.77 (1.61-1.94) | 1.33 (1.16-1.53) | 1.40 (1.25-1.56) |
| Model 3^c^ | 1.31 (0.94-1.83) | 1.26 (0.88-1.81) | 1.20 (1.04-1.39) | 0.92 (0.77-1.10) | ^e^ |

Note: CI = confidence interval, HR = hazard ratio, MI = myocardial infarction

^a^Unadjusted, baseline hazard allowed to vary across states.

^b^Adjusted for age, sex, and race/ethnicity, baseline hazard allowed to vary across states.

^c^Adjusted for age, sex, race/ethnicity, area-level median income, alcohol and tobacco use, primary versus secondary diagnosis position for initial MI, coronary revascularization during hospitalization for initial MI, history of diabetes mellitus, chronic kidney disease, stroke, heart failure, peripheral artery disease, depression, and dementia, frailty, cardiologist care, cardiac rehabilitation, any hospitalization within the past year, statin use and intensity (i.e., no statin use, use of low/moderate-intensity statins, use of high-intensity statins), non-statin lipid lowering therapy, use of beta-blocker, antiplatelet agents, prevalent nursing home residence (mortality and impoverishment outcomes), and prevalent impoverishment (mortality and long-term nursing home placement outcomes), baseline hazard allowed to vary across states.

^d^Defined as new Medicaid or Medicare Part D subsidy eligibly

^e^Not estimable because of small number of events in the recurrent MI cohort after 731 days of follow-up

**Supplementary Table 2.** Characteristics associated with death, long-term nursing home placement, and impoverishment among Medicare beneficiaries

|  | **Hazard ratio (95% confidence interval)** | | |
| --- | --- | --- | --- |
|  | **Death** | **Long-term nursing home placement** | **Impoverishment^a^** |
| Age |  |  |  |
| 66 – 70 years | 1 (reference) | 1 (reference) | 1 (reference) |
| 71 – 75 years | 1.10 (1.08-1.12) | 1.13 (1.06-1.20) | 1.05 (0.95-1.15) |
| 76 – 80 years | 1.25 (1.23-1.27) | 1.36 (1.28-1.44) | 1.09 (0.99-1.19) |
| 81 – 85 years | 1.42 (1.39-1.44) | 1.69 (1.59-1.79) | 1.20 (1.10-1.32) |
| ≥86 years | 1.81 (1.78-1.85) | 2.38 (2.25-2.51) | 1.32 (1.20-1.45) |
| Sex |  |  |  |
| Male | 1 (reference) | 1 (reference) | 1 (reference) |
| Female | 0.84 (0.84-0.85) | 0.97 (0.94-1.01) | 1.51 (1.42-1.59) |
| Race/Ethnicity |  |  |  |
| White | 1 (reference) | 1 (reference) | 1 (reference) |
| Black | 0.93 (0.91-0.95) | 0.86 (0.82-0.91) | 1.48 (1.33-1.65) |
| Hispanic | 0.88 (0.85-0.91) | 0.63 (0.57-0.70) | 1.92 (1.43-2.59) |
| Asian | 0.92 (0.89-0.96) | 0.67 (0.60-0.75) | 1.18 (0.83-1.68) |
| Other | 0.93 (0.90-0.96) | 0.79 (0.71-0.88) | 1.08 (0.84-1.38) |
| Area-level median income |  |  |  |
| <$30,000 | 1 (reference) | 1 (reference) | 1 (reference) |
| $30,000 - $44,999 | 0.99 (0.97-1.01) | 1.13 (1.06-1.20) | 0.89 (0.79-1.01) |
| $45,000 - $59,999 | 0.99 (0.97-1.01) | 1.15 (1.07-1.22) | 0.75 (0.66-0.86) |
| $60,000 - $74,999 | 0.98 (0.96-1.00) | 1.23 (1.14-1.32) | 0.63 (0.54-0.72) |
| ≥$75,000 | 0.97 (0.94-0.99) | 1.24 (1.15-1.34) | 0.53 (0.46-0.61) |
| Alcohol use | 0.88 (0.78-0.99) | 0.72 (0.50-1.04) | 0.57 (0.24-1.37) |
| Tobacco use | 1.04 (1.03-1.05) | 0.88 (0.85-0.91) | 0.87 (0.82-0.93) |
| Diagnosis position for first MI |  |  |  |
| Primary | 1 (reference) | 1 (reference) | 1 (reference) |
| Secondary | 1.06 (1.04-1.07) | 1.07 (1.03-1.10) | 0.95 (0.90-1.01) |
| Coronary revascularization during initial MI | 0.75 (0.74-0.76) | 0.79 (0.76-0.82) | 0.87 (0.82-0.93) |
| History of diabetes mellitus | 0.99 (0.98-1.00) | 0.98 (0.95-1.01) | 1.22 (1.15-1.29) |
| History of chronic kidney disease | 1.41 (1.39-1.43) | 1.01 (0.98-1.05) | 1.03 (0.98-1.09) |
| History of stroke | 1.17 (1.16-1.19) | 1.28 (1.23-1.33) | 1.22 (1.13-1.31) |
| History of heart failure | 1.57 (1.55-1.59) | 1.14 (1.10-1.19) | 1.23 (1.16-1.31) |
| History of peripheral artery disease | 1.13 (1.12-1.15) | 1.05 (1.02-1.09) | 1.09 (1.03-1.16) |
| History of depression | 1.03 (1.02-1.04) | 1.37 (1.32-1.41) | 1.21 (1.14-1.28) |
| History of dementia | 1.16 (1.14-1.17) | 1.96 (1.89-2.03) | 1.48 (1.37-1.59) |
| Frailty |  |  |  |
| Robust | 1 (reference) | 1 (reference) | 1 (reference) |
| Pre-frail | 1.37 (1.32-1.43) | 1.25 (1.10-1.43) | 1.31 (1.11-1.55) |
| Mildly frail | 1.81 (1.73-1.88) | 1.87 (1.63-2.13) | 1.83 (1.55-2.18) |
| Moderate-to-severely frail | 2.07 (1.99-2.16) | 2.37 (2.06-2.71) | 2.05 (1.71-2.45) |
| Cardiologist care | 0.88 (0.87-0.89) | 0.68 (0.66-0.70) | 0.72 (0.68-0.76) |
| Any hospitalization within the past year | 1.04 (1.02-1.05) | 0.96 (0.93-1.01) | 1.18 (1.10-1.26) |
| Statin use and intensity |  |  |  |
| No statins | 1 (reference) | 1 (reference) | 1 (reference) |
| Low to moderate intensity statins | 0.89 (0.88-0.90) | 0.91 (0.88-0.95) | 0.98 (0.92-1.04) |
| High-intensity statins | 0.82 (0.81-0.84) | 0.81 (0.78-0.85) | 0.82 (0.76-0.88) |
| Non-statin lipid lowering treatment | 0.94 (0.93-0.95) | 0.94 (0.90-0.99) | 0.89 (0.83-0.96) |
| Use of beta-blockers | 0.95 (0.93-0.96) | 0.96 (0.93-1.00) | 0.95 (0.89-1.02) |
| Use of antiplatelet agents | 0.95 (0.94-0.96) | 0.92 (0.89-0.95) | 0.93 (0.88-0.98) |
| Cardiac rehabilitation | 0.56 (0.54-0.58) | 0.67 (0.60-0.74) | 0.53 (0.47-0.60) |
| Prevalent debility | 1.11 (1.09-1.13) | -- | 1.25 (1.11-1.41) |
| Prevalent destitution | 1.05 (1.04-1.06) | 2.02 (1.95-2.09) | -- |

Note: Numbers in table are hazard ratios (95% confidence intervals) from models including all variables listed in the table, baseline hazard allowed to vary across states. MI = myocardial infarction

^a^Defined as new Medicaid or Medicare Part D subsidy eligibly

**Supplementary Table 3.** Characteristics of Medicare beneficiaries with and without recurrent myocardial infarction (MI) (subgroup with primary discharge diagnosis of MI during initial MI hospitalization)

|  | Control cohort with initial MI (n=642,993) | Recurrent MI cohort (n=154,737) |
| --- | --- | --- |
| Age, n (%) |  |  |
| 66 – 70 years | 119,137 (18.5%) | 24,707 (16.0%) |
| 71 – 75 years | 143,060 (22.2%) | 29,345 (19.0%) |
| 76 – 80 years | 130,826 (20.3%) | 29,130 (18.8%) |
| 81 – 85 years | 115,086 (17.9%) | 28,989 (18.7%) |
| ≥86 | 134,884 (21.0%) | 42,566 (27.5%) |
| Sex, n (%) |  |  |
| Male | 299,221 (46.5%) | 69,614 (45.0%) |
| Female | 343,772 (53.5%) | 85,123 (55.0%) |
| Race/Ethnicity, n (%) |  |  |
| White | 558,924 (86.9%) | 128,632 (83.1%) |
| Black | 47,037 (7.3%) | 15,682 (10.1%) |
| Hispanic | 13,340 (2.1%) | 4,047 (2.6%) |
| Asian | 10,634 (1.7%) | 3,099 (2.0%) |
| Other | 13,058 (2.0%) | 3,277 (2.1%) |
| Area-level median income, n (%) |  |  |
| Not known | 11,051 (1.7%) | 2,773 (1.8%) |
| <$30,000 | 36,118 (5.6%) | 10,690 (6.9%) |
| $30,000 - $44,999 | 227,765 (35.4%) | 56,716 (36.7%) |
| $45,000 - $59,999 | 184,540 (28.7%) | 43,447 (28.1%) |
| $60,000 - $74,999 | 90,572 (14.1%) | 20,878 (13.5%) |
| ≥$75,000 | 92,947 (14.5%) | 20,233 (13.1%) |
| Alcohol use, n (%) | 1,020 (0.2%) | 363 (0.2%) |
| Tobacco use, n (%) | 174,493 (27.1%) | 48,050 (31.1%) |
| Coronary revascularization during initial MI hospitalization, n (%) | 372,224 (57.9%) | 64,440 (41.6%) |
| History of diabetes mellitus, n (%) | 258,373 (40.2%) | 85,667 (55.4%) |
| History of chronic kidney disease, n (%) | 221,694 (34.5%) | 99,877 (64.5%) |
| History of stroke, n (%) | 52,230 (8.1%) | 21,172 (13.7%) |
| History of heart failure, n (%) | 257,418 (40.0%) | 112,706 (72.8%) |
| History of peripheral artery disease, n (%) | 90,498 (14.1%) | 41,819 (27.0%) |
| History of depression, n (%) | 211,178 (32.8%) | 60,428 (39.1%) |
| History of dementia, n (%) | 74,828 (11.6%) | 28,570 (18.5%) |
| Frailty, n (%) |  |  |
| Robust | 59,566 (9.3%) | 5,097 (3.3%) |
| Pre-frail | 299,699 (46.6%) | 52,405 (33.9%) |
| Mildly frail | 181,414 (28.2%) | 56,218 (36.3%) |
| Moderate-to-severely frail | 102,314 (15.9%) | 41,017 (26.5%) |
| Cardiologist care, n (%) | 423,986 (65.9%) | 97,428 (63.0%) |
| Any hospitalization within the past year, n (%) | 429,503 (66.8%) | 116,837 (75.5%) |
| Statin use and intensity, n (%) |  |  |
| No statins | 130,438 (20.3%) | 36,732 (23.7%) |
| Low to moderate intensity statins | 356,922 (55.5%) | 75,610 (48.9%) |
| High-intensity statins | 155,633 (24.2%) | 42,395 (27.4%) |
| Non-statin lipid lowering treatment, n (%) | 81,982 (12.8%) | 20,990 (13.6%) |
| Use of beta-blockers, n (%) | 533,777 (83.0%) | 129,678 (83.8%) |
| Use of antiplatelet agents, n (%) | 356,162 (55.4%) | 86,902 (56.2%) |
| Cardiac rehabilitation, n (%) | 28,534 (4.4%) | 10,012 (6.5%) |
| Prevalent nursing home residence, n (%) | 34,326 (5.3%) | 9,238 (6.0%) |
| Prevalent impoverishment, n (%) | 234,527 (36.5%) | 71,983 (46.5%) |

Note: MI = myocardial infarction

**Supplementary Table 4.** Association of recurrent MI with death, long-term nursing home placement, and impoverishment among Medicare beneficiaries (subgroup with primary discharge diagnosis of MI during initial MI hospitalization)

|  | **Control cohort with initial MI** | **Recurrent MI cohort** |
| --- | --- | --- |
| *Death* |  |  |
| Event/person-years | 253,666/1,939,888 | 104,464/263,632 |
| Rate per 1,000 person-years (95% CI) | 130.8 (130.3-131.3) | 396.2 (393.9-398.7) |
| HR (95% CI) |  |  |
| Model 1^a^ | 1 (reference) | 2.83 (2.81-2.85) |
| Model 2^b^ | 1 (reference) | 2.71 (2.69-2.73) |
| Model 3^c^ | 1 (reference) | 2.01 (2.00-2.03) |
| *Long-term nursing home placement* |  |  |
| Event/person-years | 49,851/1,786,824 | 11,112/240,915 |
| Rate per 1,000 person-years (95% CI) | 27.9 (27.7-28.1) | 46.1 (45.3-47.0) |
| HR (95% CI) |  |  |
| Model 1^a^ | 1 (reference) | 1.27 (1.25-1.30) |
| Model 2^b^ | 1 (reference) | 1.17 (1.15-1.20) |
| Model 3^c^ | 1 (reference) | 0.91 (0.89-0.93) |
| *Impoverishment*^d^ |  |  |
| Event/person-years | 21,260/1,244,208 | 4,252/143,347 |
| Rate per 1,000 person-years (95% CI) | 17.1 (16.9-17.3) | 29.7 (28.8-30.6) |
| HR (95% CI) |  |  |
| Model 1^a^ | 1 (reference) | 1.65 (1.59-1.70) |
| Model 2^b^ | 1 (reference) | 1.55 (1.50-1.60) |
| Model 3^c^ | 1 (reference) | 1.32 (1.27-1.36) |

Note: CI = confidence interval, HR = hazard ratio, MI= myocardial infarction

^a^Unadjusted, baseline hazard allowed to vary across states.

^b^Adjusted for age, sex, and race/ethnicity, baseline hazard allowed to vary across states.

^c^Adjusted for age, sex, race/ethnicity, area-level median income, alcohol and tobacco use, coronary revascularization during hospitalization for initial MI, history of diabetes mellitus, chronic kidney disease, stroke, heart failure, peripheral artery disease, depression, and dementia, frailty, cardiologist care, cardiac rehabilitation, any hospitalization within the past year, statin use and intensity (i.e., no statin use, use of low/moderate-intensity statins, use of high-intensity statins), non-statin lipid lowering therapy, use of beta-blocker, antiplatelet agents, prevalent nursing home residence (mortality and impoverishment outcomes), and prevalent impoverishment (mortality and long-term nursing home placement outcomes), baseline hazard allowed to vary across states.

^d^Defined as new Medicaid or Medicare Part D subsidy eligibly

**Supplementary Table 5.** Characteristics associated with death, long-term nursing home placement, and impoverishment among Medicare beneficiaries with recurrent MI (subgroup with primary discharge diagnosis of MI during initial MI hospitalization)

|  | **Death** | **Long-term nursing home placement** | **Impoverishment**^a^ |
| --- | --- | --- | --- |
| Age |  |  |  |
| 66 – 70 years | 1 (reference) | 1 (reference) | 1 (reference) |
| 71 – 75 years | 1.10 (1.08-1.13) | 1.13 (1.04-1.22) | 1.01 (0.90-1.13) |
| 76 – 80 years | 1.27 (1.24-1.30) | 1.45 (1.34-1.57) | 1.07 (0.95-1.20) |
| 81 – 85 years | 1.46 (1.43-1.49) | 1.82 (1.68-1.96) | 1.23 (1.10-1.38) |
| ≥86 years | 1.88 (1.84-1.93) | 2.62 (2.43-2.81) | 1.37 (1.22-1.53) |
| Sex |  |  |  |
| Male | 1 (reference) | 1 (reference) | 1 (reference) |
| Female | 0.85 (0.83-0.86) | 0.98 (0.94-1.02) | 1.49 (1.39-1.59) |
| Race/Ethnicity |  |  |  |
| White | 1 (reference) | 1 (reference) | 1 (reference) |
| Black | 0.92 (0.90-0.94) | 0.82 (0.77-0.88) | 1.51 (1.33-1.72) |
| Hispanic | 0.86 (0.83-0.89) | 0.61 (0.54-0.70) | 2.12 (1.52-2.96) |
| Asian | 0.93 (0.89-0.97) | 0.68 (0.59-0.79) | 1.09 (0.70-1.70) |
| Other | 0.92 (0.88-0.96) | 0.86 (0.75-0.98) | 1.01 (0.74-1.37) |
| Area-level median income |  |  |  |
| <$30,000 | 1 (reference) | 1 (reference) | 1 (reference) |
| $30,000 - $44,999 | 1.00 (0.98-1.03) | 1.16 (1.07-1.25) | 0.88 (0.75-1.02) |
| $45,000 - $59,999 | 1.00 (0.97-1.03) | 1.15 (1.06-1.25) | 0.73 (0.63-0.86) |
| $60,000 - $74,999 | 1.00 (0.97-1.03) | 1.27 (1.16-1.40) | 0.61 (0.52-0.73) |
| ≥$75,000 | 0.97 (0.94-1.00) | 1.25 (1.13-1.37) | 0.52 (0.44-0.61) |
| Alcohol use | 0.84 (0.72-0.98) | 0.77 (0.47-1.27) | 1.04 (0.43-2.51) |
| Tobacco use | 1.04 (1.02-1.05) | 0.90 (0.86-0.94) | 0.86 (0.80-0.93) |
| Coronary revascularization during initial MI hospitalization | 0.75 (0.74-0.76) | 0.80 (0.76-0.83) | 0.88 (0.82-0.94) |
| History of diabetes mellitus | 1.00 (0.99-1.02) | 0.97 (0.94-1.01) | 1.24 (1.16-1.33) |
| History of chronic kidney disease | 1.45 (1.43-1.47) | 1.04 (1.00-1.08) | 1.05 (0.99-1.13) |
| History of stroke | 1.20 (1.18-1.22) | 1.28 (1.22-1.34) | 1.19 (1.09-1.31) |
| History of heart failure | 1.67 (1.64-1.70) | 1.17 (1.12-1.23) | 1.22 (1.13-1.31) |
| History of peripheral artery disease | 1.15 (1.13-1.16) | 1.04 (1.00-1.09) | 1.10 (1.02-1.18) |
| History of depression | 1.04 (1.03-1.06) | 1.37 (1.32-1.43) | 1.18 (1.11-1.26) |
| History of dementia | 1.16 (1.14-1.18) | 1.98 (1.89-2.07) | 1.57 (1.44-1.71) |
| Frailty |  |  |  |
| Robust | 1 (reference) | 1 (reference) | 1 (reference) |
| Pre-frail | 1.40 (1.34-1.47) | 1.30 (1.11-1.51) | 1.36 (1.13-1.64) |
| Mildly frail | 1.86 (1.77-1.95) | 1.88 (1.61-2.20) | 1.95 (1.60-2.37) |
| Moderate-to-severely frail | 2.13 (2.02-2.23) | 2.41 (2.05-2.83) | 2.20 (1.78-2.71) |
| Cardiologist care | 0.87 (0.86-0.89) | 0.69 (0.66-0.71) | 0.75 (0.70-0.80) |
| Any hospitalization within the past year | 1.03 (1.02-1.05) | 0.99 (0.94-1.05) | 1.17 (1.08-1.27) |
| Statin use and intensity |  |  |  |
| No statins | 1 (reference) | 1 (reference) | 1 (reference) |
| Low to moderate intensity statins | 0.89 (0.88-0.90) | 0.93 (0.89-0.97) | 0.95 (0.88-1.03) |
| High-intensity statins | 0.82 (0.81-0.84) | 0.82 (0.78-0.87) | 0.81 (0.74-0.89) |
| Non-statin lipid lowering treatment | 0.94 (0.92-0.96) | 0.95 (0.90-1.01) | 0.89 (0.81-0.97) |
| Use of beta-blockers | 0.95 (0.94-0.97) | 0.97 (0.92-1.02) | 0.92 (0.85-1.00) |
| Use of antiplatelet agents | 0.95 (0.94-0.96) | 0.92 (0.89-0.96) | 0.92 (0.86-0.98) |
| Cardiac rehabilitation | 0.57 (0.56-0.59) | 0.65 (0.58-0.73) | 0.52 (0.46-0.60) |
| Prevalent nursing home residence | 1.12 (1.10-1.15) | -- | 1.30 (1.12-1.50) |
| Prevalent impoverishment | 1.05 (1.04-1.07) | 1.96 (1.88-2.05) | -- |

Note: numbers in table are hazard ratios (95% confidence intervals) from models including all variables listed in the table, baseline hazard allowed to vary across states. MI = myocardial infarction

^a^Defined as new Medicaid or Medicare Part D subsidy eligibly
